# Chemotherapy-Induced Cardiotoxicity: Mechanisms, Detection and Emerging Therapies in Cardio-Oncology

**DOI:** 10.15190/d.2025.16

**Published:** 2025-12-31

**Authors:** Brandon Sánchez, Pamela González, Iván Goveo, Pedro Contreras, Luis Domínguez, Paola Manrique, Manuel Vargas

**Affiliations:** ^1^Pontificia Universidad Católica Madre y Maestra. Santiago, Dominican Republic; ^2^Universidad Autonoma de Guadalajara, Guadalajara, Mexico; ^3^Corazones del Cibao, Santiago, Dominican Republic

**Keywords:** Cardio-oncology, chemotherapy-induced heart failure, cardiotoxicity, anthracyclines, biomarkers, cardioprotection.

## Abstract

Cancer remains a leading cause of global mortality, with annual incidence projected to exceed 35 million cases by 2050. Modern antineoplastic therapies have improved survival outcomes at the risk of increasingly associated cardiovascular complications, collectively termed cancer therapy related cardiac dysfunction (CTRCD). Anthracyclines and HER2-targeted therapies remain the most well-characterized cardiotoxic agents. Anthracyclines cause irreversible, dose-dependent myocardial injury through mechanisms including oxidative stress, iron dysregulation, mitochondrial dysfunction, and topoisomerase IIβ inhibition, leading to progressive ventricular dysfunction and heart failure. HER2-directed therapies, such as trastuzumab, interfere with cardioprotective ErbB signaling, typically producing reversible cardiac impairment. Other oncologic treatments - including tyrosine kinase inhibitors, VEGF antagonists, and immune checkpoint inhibitors - contribute to hypertension, ischemic injury, and immune-mediated myocarditis. Newer modalities, such as proteasome inhibitors, histone deacetylase inhibitors, and CAR T-cell therapy, have expanded the spectrum of treatment-associated cardiotoxicity. Early CTRCD detection through multimodal strategies - including echocardiographic assessment with global longitudinal strain, cardiac magnetic resonance imaging, and serial measurement of troponins and natriuretic peptides - facilitates timely intervention. Risk stratification tools such as the HFA–ICOS score enable personalized monitoring and therapeutic planning. Preventive and management strategies incorporate cardioprotective agents like ACE inhibitors, β-blockers, dexrazoxane, and emerging therapies such as SGLT2 inhibitors. Modern cardio-oncology emphasizes a multidisciplinary, precision-based approach integrating early detection, genetic risk assessment, and targeted prophylaxis to preserve cardiac function while maintaining oncologic efficacy, thereby enhancing both survival and quality of life for cancer patients.

## SUMMARY

1. Introduction

2. Epidemiology

3. Risk Factors for Cancer Therapy-Related Cardiac Dysfunction

4. Mechanisms of Cardiotoxicity

- 4.1 Mechanisms of Anthracycline-Induced Cardiotoxicity

- 4.2 Mechanisms of Trastuzumab-Induced Cardiotoxicity

- 4.3 Cardiotoxic Mechanisms of Targeted and Immune-Based Therapies

5. Detection and Monitoring

- 5.1 Imaging

- 5.2 Biomarkers

6. Prevention and Management

7. Emerging Directions

8. Conclusion

## 1. Introduction

Cancer represents one of the leading causes of death worldwide and imposes a considerable global disease burden^1^. According to the World Health Organization, in 2022, approximately 20 million new cancer cases and 9.7 million deaths were reported globally, and the incidence is expected to rise to over 35 million by 2050^2^. The use of antineoplastic agents has intensified as part of therapeutic strategies for these diseases, contributing to reduced mortality and increased cancer survival^3^. However, these treatments can cause significant adverse effects, including cardiotoxicity^4^. Among these drugs, anthracyclines, introduced in 1960, have transformed the management of both solid and hematologic malignancies and remain essential in lymphomas, acute leukemias, and sarcomas. Their use, however, is limited by dose-dependent cardiotoxicity, which can impair cardiac function and lead to heart failure^5^. Similarly, chemotherapeutic agents like anthracyclines induce damage by generating reactive oxygen species (ROS), resulting in oxidative stress and irreversible injury to cardiomyocytes^6^.

Furthermore, therapies targeting the vascular endothelial growth factor (VEGF) pathway, used to treat solid tumors, can lead to cardiovascular effects such as hypertension and myocardial hypoperfusion, contributing to the development of heart failure. With the advent of more potent medications, patients often face the dual challenge of controlling cancer while simultaneously protecting cardiovascular health^7^. Therefore, the 2022 European Society of Cardiology (ESC) cardio-oncology guidelines emphasize the importance of a multidisciplinary and personalized approach to safeguard cardiovascular health throughout oncologic treatment^8^.

In this context, early detection of cardiotoxicity using biomarkers such as troponins and imaging techniques, including echocardiography and cardiac magnetic resonance (CMR), allows identification of cardiovascular risks before irreversible damage occurs. Prevention and management strategies include the early use of cardioprotective drugs, such as beta-blockers, angiotensin-converting enzyme (ACE) inhibitors, and angiotensin II receptor antagonists, which help mitigate cardiovascular side effects of oncologic therapy and enable patients to continue potentially life-saving treatments. Cardio-oncology thus aims to optimize cancer care while protecting the heart, improving both survival and quality of life for patients^7^. While recent high-impact guidelines, provide comprehensive frameworks for clinical risk stratification and surveillance strategies, the present review offers a distinct contribution by integrating mechanistic depth with emerging precision medicine approaches. Specifically, this manuscript bridges molecular pathophysiology with clinical application by synthesizing the latest evidence on subcellular mechanisms of cardiotoxicity across traditional and novel therapeutics, while emphasizing the evolving role of pharmacogenetic risk assessment and personalized cardioprotective strategies

## **2. **Epidemiology

Oncologic therapies can impair cardiac structure and/or function, manifesting either as asymptomatic cardiac dysfunction or symptomatic heart failure, collectively termed cancer therapy–related cardiac dysfunction (CTRCD)^9^. The risk of cardiotoxicity in cancer patients is influenced by multiple factors, including the specific chemotherapeutic agent used, cumulative dose, combination regimens, and patient-specific characteristics such as age, pre-existing cardiovascular disease, and genetic susceptibility. Likewise, the prevalence of late-onset symptomatic cardiotoxicity associated with anthracyclines is affected by the type of cancer treated, concomitant cardiovascular risk factors, baseline cardiac conditions, and the duration of follow-up^7^.

Anthracyclines, including doxorubicin, daunorubicin, epirubicin, and idarubicin, are commonly employed for a variety of malignancies, including breast cancer and lymphoma. Their use is associated with dose-dependent cardiomyocyte injury and death, potentially leading to left ventricular dysfunction and heart failure. In three studies involving 630 patients with breast and lung cancer, the incidence of clinical heart failure during doxorubicin therapy rose exponentially, from 5% at a cumulative dose of 400 mg/m² to 48% at 700 mg/m² ^10^. When asymptomatic reductions in left ventricular ejection fraction (LVEF) were included in the cardiotoxicity definition, the rate of cardiac events increased substantially, even at lower cumulative doses: 7%, 18%, and 65% with 150 mg/m², 350 mg/m², and 550 mg/m², respectively^10^.

A meta-analysis of 53 studies encompassing 35,651 patients estimated a pooled overall incidence of chemotherapy-related cardiac dysfunction of 63.21 per 1000 person-years (95% CI: 57.28–69.14). Incidence rose sharply within the first six months of chemotherapy initiation and stabilized with longer follow-up. Stratified analyses indicated a markedly higher incidence in patients aged ≥50 years compared with those <50 years (99.96 vs. 34.48 per 1000 person-years). By cancer type, the highest incidence was observed in patients with breast cancer (72.97 per 1000 person-years), followed by leukemia (65.21 per 1000 person-years) and lymphoma (55.43 per 1000 person-years)^11^.

## 3. Risk Factors for Cancer Therapy-Related Cardiac Dysfunction

The risk of CTRCD varies considerably among patients, and much of this variation is influenced by their baseline cardiovascular health. Age remains one of the most important risk factors. Older adults typically have reduced cardiac reserve, increased vascular stiffness, and a higher prevalence of underlying heart disease, all of which make them more susceptible to the cardiotoxic effects of cancer treatments^12^. In addition, comorbid conditions such as hypertension, diabetes, obesity, and coronary artery disease further elevate the likelihood of cardiotoxicity by imposing additional stress on the myocardium and vascular system. Recent meta-analyses consistently demonstrate that patients with these pre-existing conditions are at a significantly higher risk of developing heart failure and other cardiovascular complications during and after cancer therapy^13^.

Genetic predisposition is increasingly recognized as an important factor influencing the risk of cardiotoxicity. Patients with similar clinical profiles can experience widely different degrees of myocardial injury, in part due to genetic polymorphisms that affect how cardiomyocytes respond to chemotherapy. For example, certain non-coding genetic variants have been associated with differences in sensitivity to anthracyclines in induced pluripotent stem cells derived from cardiomyocytes, highlighting the role of heritable factors in CTRCD susceptibility^8^. In addition to genetic influences, modifiable lifestyle factors such as tobacco use, physical inactivity, previous myocarditis, and pre-existing heart failure can further increase cardiac risk and should be addressed before initiating cancer therapy^14^. Incorporating these variables including age, comorbid conditions, genetic background, and lifestyle behaviors into structured pre-treatment assessments, such as the Heart Failure Association–International Cardio-Oncology Society (HFA-ICOS) risk score, allows clinicians to better identify high-risk patients and to design individualized surveillance and prevention strategies.

Beyond patient characteristics, the type and delivery of cancer therapy itself strongly influence the risk of cardiotoxicity. Anthracyclines remain one of the leading causes of CTRCD, with a well-established dose-dependent relationship between cumulative exposure and the likelihood of left ventricular dysfunction^15^. HER2-targeted therapies, such as trastuzumab, are inherently less cardiotoxic but can significantly increase the risk of cardiac injury when administered concurrently or sequentially with anthracyclines, creating a well-recognized “two-hit” phenomenon^16^. This underscores the importance of careful treatment sequencing and the need for intensified cardiac monitoring in patients receiving both agents.

Dose and infusion schedule also influence cardiotoxic risk. Rapid bolus administration and higher cumulative doses are associated with more pronounced myocardial injury. Advanced imaging techniques, such as global longitudinal strain (GLS), can detect early, subclinical changes in cardiac function, allowing for timely intervention before significant ventricular dysfunction occurs^14^. Thoracic radiation therapy adds another layer of complexity. Contemporary evidence shows that higher mean heart doses and greater radiation exposure to coronary structures significantly increase the risk of ischemic events and heart failure, particularly when combined with prior anthracycline exposure^17^. Efforts to reduce cardiac radiation including optimized treatment planning, fractionation strategies, and heart-sparing techniques, are essential for risk mitigation.

Finally, targeted therapiesandtyrosine kinase inhibitors (TKIs) present additional cardiovascular risks, including hypertension, myocardial ischemia, arrhythmias, and heart failure. The HFA-ICOS risk score incorporates drug class, cumulative exposure, and combination treatment effects into overall risk assessment, enabling clinicians to adjust surveillance frequency and implement cardioprotective strategies, such as ACE inhibitors or beta-blockers, when appropriate^8^.

## 4. Mechanisms of Cardiotoxicity

### 4.1 Mechanisms of Anthracycline-Induced Cardiotoxicity

The therapeutic efficacy of anthracyclines stems from multiple cellular mechanisms that result in both cytotoxic and growth-inhibitory effects. The predominant mechanism involves anthracycline binding to topoisomerase II, creating a triple molecular complex consisting of topoisomerase II, doxorubicin, and DNA. This complex disrupts the normal DNA repair process by blocking the rejoining of cleaved double-strand DNA breaks^18^. The consequence is cell cycle arrest followed by programmed cell death. Two distinct forms of topoisomerase II exist: the alpha (α) and beta (β) isoforms. The alpha isoform shows elevated expression levels in cancer cells, accounting for the preferential anticancer activity of anthracycline treatment^19^. Furthermore, anthracyclines can insert themselves between DNA base pairs within the nuclear compartment, inhibiting DNA and RNA synthesis^20^. Anthracyclines also alter gene expression patterns, impairing cellular biogenesis and function. Through topoisomerase-IIβ–dependent mechanisms, they suppress the expression of protective antioxidant enzymes, which further amplifies reactive oxygen species production. Together, these changes drive oxidative damage within malignant tissues^21,22^. However, among the significant adverse effects of chemotherapy, drug-induced cardiotoxicity represents a critical concern requiring careful monitoring. The cardiovascular manifestations encompass a wide range of clinical presentations, most commonly presenting as dose-dependent left ventricular dysfunction, which may progress to heart failure. Arrhythmias and pericarditis are less frequent but clinically relevant manifestations^23-25^.

While the precise mechanisms underlying anthracycline-induced cardiac damage remain elusive, multiple pathological processes have been identified. Among these, anthracycline-mediated suppression of topoisomerase-II β (Top2β) in cardiac muscle cells and the generation of reactive oxygen species are now widely accepted as central mechanisms of anthracycline-induced cardiotoxicity^18^. Zhang and colleagues demonstrated in 2012 that the elimination of topoisomerase-II β provides cardiac protection against doxorubicin-induced DNA strand breaks. This mechanism has subsequently been confirmed as pivotal and widely accepted in understanding doxorubicin-related cardiomyocyte injury^22,26^. Their findings suggested that Top2β plays a central role in doxorubicin-related heart muscle damage. Mouse models lacking Top2β showed a marked reduction in cardiac cell death following doxorubicin exposure, supporting the significance of Top2β-anthracycline interactions in disease development^22^. The binding of anthracyclines to Top2β in heart muscle cells triggers double-strand DNA damage, which subsequently activates p53-dependent cell death pathways^27^.

**Figure 1:**
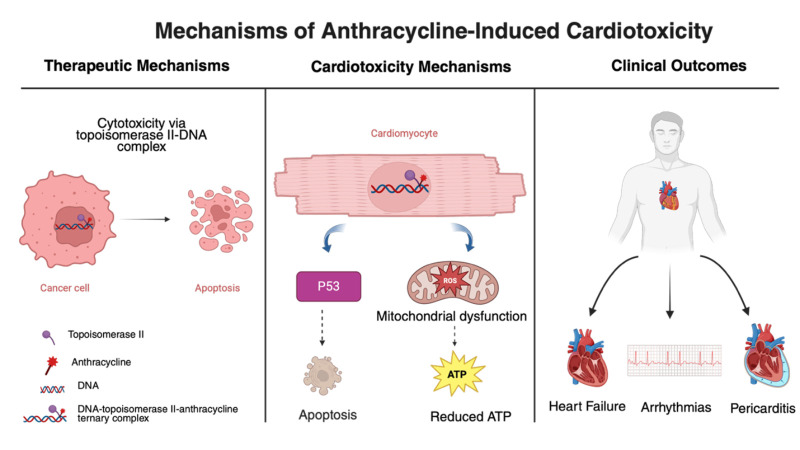
Figure 1. Pathways of Cardiac Damage from Anthracycline Chemotherapy. Anthracyclines achieve their cancer-fighting activity by creating a three-component structure with topoisomerase IIα and DNA, resulting in breaks in both DNA strands and subsequent death of malignant cells. Within cardiac muscle cells, interaction with topoisomerase IIβ initiates genetic damage and cell death mediated by p53. At the same time, anthracyclines cause iron metabolism dysregulation, which enhances the production of reactive oxygen species, causing damage to mitochondria, a reduction in ATP availability, and disruption of calcium balance. Damage to the inner lining of blood vessels and compromised vascular communication additionally promotes oxygen deficiency in tissues. Together, these pathways lead to ongoing loss of cardiac muscle cells and deterioration of left ventricular function, resulting in clinical manifestations ranging from heart failure, arrhythmias to pericarditis (Created with BioRender.com)^21,28-32^

Recent medical literature robustly supports the multifactorial mechanisms underlying anthracycline-induced cardiac damage, extending well beyond the classic pathways of DNA intercalation and topoisomerase II inhibition. Disruption of cellular iron regulation is a key contributor, with anthracyclines causing mitochondrial iron accumulation, which exacerbates reactive oxygen species formation and mitochondrial dysfunction ^28-30^. Reduced production of energy molecules, particularly ATP, results from anthracycline-induced suppression of mitochondrial respiratory chain activity and persistent defects in high-energy phosphate metabolism, which have been documented years after exposure ^29,31^. Blood vessel dysfunction is increasingly recognized as a contributor to anthracycline cardiotoxicity, with evidence of endothelial injury and impaired vascular signaling ^28,31^. Altered calcium handling mechanisms, such as changes in mitochondrial membrane permeability and sarcoplasmic reticulum dilation, disrupt intracellular calcium homeostasis and impair contractility ^31,32^. Collectively, these mechanisms—iron dysregulation, mitochondrial injury, energy depletion, protein degradation, vascular dysfunction, and calcium mishandling—interact to drive myocyte death and progressive contractile dysfunction, as consistently described in recent reviews and scientific statements (see **Figure 1**)^21,28-32^.

### 4.2 Mechanisms of Trastuzumab-Induced Cardiotoxicity

HER2 is a transmembrane glycoprotein possessing intrinsic tyrosine kinase activity and belongs to the epidermal growth factor receptor (EGFR) family. This protein participates in intracellular signaling pathways that regulate cellular growth, survival, adhesion, migration, and proliferation^33^. Trastuzumab, a HER2-directed monoclonal antibody, represents the standard therapeutic approach for HER2-positive malignancies. The mechanism of

action involves inhibition of HER2-positive tumor cell growth through disruption of growth factor-tumor cell interactions^34^.

Cardiotoxicity represents the most frequently observed adverse effect associated with trastuzumab therapy, presenting clinically as decreased left ventricular ejection fraction, cardiac arrhythmias, or congestive heart failure, occurring independently of dosage^35,36^. In contrast to anthracycline-induced cardiotoxicity, trastuzumab-related cardiac dysfunction demonstrates reversibility due to the absence of ultrastructural cardiomyocyte alterations ^35^.

The pathophysiological mechanisms underlying trastuzumab-induced cardiotoxicity remain incompletely elucidated, though several hypotheses have been proposed. Given trastuzumab's nature as a monoclonal antibody, antibody-dependent cellular cytotoxicity may contribute to cardiomyocyte injury^36^. Within cardiac tissue, HER4 interacts with the erythroblastic oncogene B2 receptor (ErbB) through heterodimer formation, subsequently activating downstream signaling cascades including phosphatidylinositol 3-kinase (PI3K)/protein kinase B (AKT), mitogen-activated protein kinase (MAPK), and endothelial nitric oxide synthase (eNOS) pathways. These signaling mechanisms promote cellular survival and proliferation, preserve mitochondrial function, and facilitate sarcoplasmic reticulum calcium handling, thereby conferring cardioprotective effects through ErbB2 activation^37^.

Trastuzumab administration results in HER2 inhibition and disruption of normal ErbB signaling through interference with Her/ErbB family dimer formation, consequently compromising cardiomyocyte survival, mitochondrial function, and sarcoplasmic reticulum calcium uptake^37^. An additional proposed mechanism involves reactive oxygen species (ROS) generation secondary to eNOS downregulation following HER2 suppression, leading to diminished antioxidant capacity and increased ROS accumulation with resultant mitochondrial damage^37,38^. Progressive mitochondrial dysfunction ultimately results in impaired cardiomyocyte contractility and cellular injury.

### 4.3 Cardiotoxic Mechanisms of Targeted and Immune-Based Therapies

**Figure 2:**
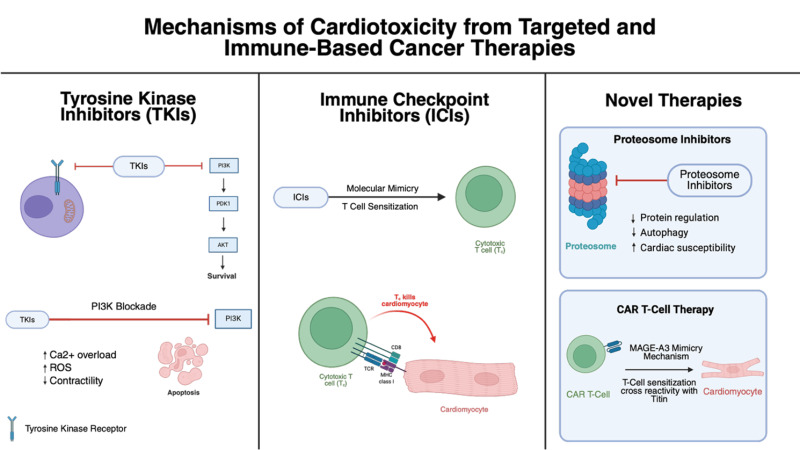
Figure 2. Pathways of Heart Damage Caused by Targeted and Immunotherapy Cancer Treatments Tyrosine kinase inhibitors (TKIs) disrupt PI3K (phosphatidylinositol 3-kinase) signaling, resulting in excessive calcium accumulation, elevated reactive oxygen species production, and compromised cardiac contractility. Immune checkpoint inhibitors (ICIs) trigger immune-driven inflammation of the heart muscle through T-cell recognition of cardiac antigens and inflammatory cytokine production. Emerging therapeutic agents, such as proteasome inhibitors, CAR T-cell immunotherapy, and histone deacetylase (HDAC) inhibitors, induce cardiac damage via disrupted protein quality control, unintended immune-mediated cardiac tissue injury, and altered gene regulation patterns. Together, these pathophysiological processes lead to impaired left ventricular function, cardiac rhythm disturbances, and heart failure development, highlighting the critical need for cardiac monitoring in the cardio-oncology management of cancer patients. (Created with BioRender.com)

The introduction of numerous immunotherapy treatments represents a significant advancement in managing various cancer types^39^. However, the cardiovascular complications associated with these precision therapies elevate the risks for individuals who survive cancer^40^. Cardiac complications related to TKIs, immune checkpoint inhibitors (ICIs), and novel targeted medications are gaining increased recognition and vary based on the therapeutic class and individual agent. Commonly reported cardiovascular adverse effects include elevated blood pressure, irregular heart rhythms, reduced heart pumping capacity, cardiac insufficiency, and abrupt cardiac death. TKIs are often linked with several cardiovascular complications. Numerous TKIs, such as sunitinib, imatinib, and trametinib, cause heart damage through a series of events that are triggered by PI3K blockade^41^. This blockade disrupts PI3K-controlled management of calcium balance, which results in excessive cytoplasmic Ca2+ accumulation, elevated ROS, and decreased contractile function (see **Figure 2**)^32,42^. Notably, several recent reports have suggested that ponatinib is the most cardiotoxic agent in all FDA-approved TKIs^43^.

Additionally, TKIs may be administered with VEGF inhibitors. VEGF is essential for angiogenesis, the development of new blood vessels, by stimulating endothelial cell activity. In cancer cells, VEGF stimulates dysregulated angiogenesis, promoting tumor growth, proliferation, and spread^44^. However, VEGF inhibitors also decrease eNOS and promote endothelin-1 production; together, these effects may lead to systemic and pulmonary hypertension, resulting in left ventricular (LV) hypertrophy and myocardial ischemia^44-46^. In animal models, endothelin-1 antagonism reversed the acute hypertension caused by VEGF inhibitors, suggesting the process may be reversible^47^. Furthermore, VEGF-TKI therapy may cause cardiotoxicity through mitochondrial dysfunction^44^. While VEGF inhibition represents a valuable anticancer strategy, its cardiovascular complications, including hypertension, ventricular remodeling, and mitochondrial damage, necessitate careful monitoring and consideration of cardioprotective interventions in clinical practice.

ICIs are associated with immune-mediated myocarditis; incidence is low but carries high mortality^48^. ICIs not only cause myocarditis, which is the most recognized cardiovascular event, but also other immune-related cardiovascular adverse events such as pericardial disease, vasculitis, acute coronary syndromes, and thromboembolic events, as well as

arrhythmias and LV dysfunction, including takotsubo syndrome, have also been described^48^. The main hypothesis centers on molecular mimicry, in which CD8+ T cells erroneously assault cardiac muscle since heart proteins resemble cancer proteins. Identical T-cell groups have been detected in malignancies, skeletal muscle, and cardiac tissue^42,48^.

Novel therapeutic agents, including proteasome inhibitors, immunomodulatory drugs, CAR T-cell therapies, and histone deacetylase inhibitors, are associated with cardiac toxicity. Research indicates that the proteasome plays an essential role in cardiac function, and its inhibition leads to ventricular dysfunction. Additionally, it disrupts key processes such as protein regulation and cell survival. Suppression of proteasome AMPKα activity decreases autophagy, resulting in left ventricular dysfunction and heightened cardiac susceptibility^49^. Carfilzomib, a proteasome inhibitor, induces myocardial complications primarily through increased PP2a activity^50^.

Another oncologic treatment, chimeric-antigen receptor (CAR) T-cell therapy (CAR-T), functions by reprogramming a patient's T lymphocytes to enhance their ability to identify and eliminate tumor cells^51^. CAR T-cell therapy can result in cardiovascular toxicities through various mechanisms, some potentially hypothetical, though several relevant theories exist. One study utilizing modified T-cell receptors (TCR) targeting the tumor antigen MAGE-A3 reported two patient deaths from cardiogenic shock and cardiac failure^52^. Autopsy findings revealed myocarditis characterized by T lymphocyte infiltration and myocyte necrosis in cardiac tissue. Notably, MAGE-A3 is not expressed in cardiac tissue; instead, the modified TCRs erroneously recognized titin, a structural protein of cardiac muscle, causing the lymphocytes to attack the heart as if it were tumor tissue^53^.

Histone deacetylase (HDAC) inhibitors similarly pose cardiac toxicity risks. HDACs regulate the genetic expression of molecules involved in oxidative stress, apoptosis, proliferation, DNA damage response, and cardiac remodeling processes that can be disrupted by HDAC inhibitors^54^. Collectively, these emerging therapies demonstrate that while they offer promising anti-cancer efficacy, their diverse mechanisms of cardiac injury, ranging from direct cellular damage to off-target immune recognition, underscore the critical need for enhanced cardio-oncology surveillance and the development of cardioprotective strategies in patients receiving these novel treatments.

## **5.** Detection and Monitoring

Early detection of CTRCD is essential to prevent progression to overt heart failure^55^. CTRCD includes both symptomatic heart failure and asymptomatic left ventricular (LV) dysfunction in patients receiving cardiotoxic treatments, most often anthracyclines and HER2-targeted agents^56^. Asymptomatic CTRCD is usually defined as an absolute LVEF decrease greater than 10 percentage points to a value below 53%^56^.

More recently, a relative GLS decrease greater than 15% from baseline and elevated cardiac biomarkers have been recognized as early signs of myocardial injury, enabling diagnosis before evident LVEF decline^57^. Baseline cardiovascular evaluation is recommended for all patients before initiating potentially cardiotoxic treatments^55^. This includes a detailed history, cardiovascular risk factor assessment, physical examination, and baseline electrocardiogram (ECG).

The HFA-ICOS cardio-oncology tools, developed in 2020, provide a comprehensive framework for stratifying cardiovascular risk in oncology patients prior to potentially cardiotoxic cancer therapies. The assessment integrates multiple factors, including the planned treatment regimen, prior cardiotoxic therapy exposure, and pre-existing cardiovascular disease. Additional considerations encompass cardiac biomarkers, patient age, traditional cardiovascular risk factors (diabetes, hypertension, chronic kidney disease), and modifiable lifestyle factors such as smoking and obesity^8^. This systematic approach enables clinicians to identify high-risk patients who may benefit from enhanced cardiovascular monitoring and preventive interventions throughout their cancer treatment.

### 5.1 Imaging

Transthoracic echocardiography (TTE) is the first-line imaging modality, given its availability, safety, and reproducibility, with strong evidence from studies of anthracycline and HER2-targeted therapy recipients^56^. Baseline TTE should include both LVEF and Global Longitudinal Strain (GLS) where feasible, as baseline LVEF and strain values are predictive of future HF risk—patients with lower-normal baseline LVEF or impaired GLS are at greater risk for CTRCD^56,58^.

During treatment, risk-stratified surveillance is recommended. High-risk patients—such as those receiving cumulative anthracycline doses ≥250 mg/m², combination anthracycline + trastuzumab, or with pre-existing cardiovascular disease- should undergo repeat TTE with GLS every 3 months. Moderate-risk patients can be monitored every 6–12 months, and long-term survivors can be monitored every 2–5 years^55,56^. A relative GLS decline of >15% or an absolute LVEF drop of ≥10% to <50% should trigger repeat imaging, initiation of cardioprotective therapy (ACE inhibitor or β-blocker), and multidisciplinary discussion regarding chemotherapy continuation^55^.

CMR is the preferred second-line modality when echocardiographic images are inconclusive or when additional tissue characterization is needed. In addition to accurate volumetric assessment, CMR provides T1/T2 mapping and late gadolinium enhancement (LGE) to detect diffuse fibrosis, edema, and inflammation-key in diagnosing myocarditis, infiltrative cardiomyopathies, and early subclinical disease^56^.

Other modalities may be used selectively. Cardiac computed tomography angiography (CCTA) provides a noninvasive assessment of coronary anatomy when invasive angiography is contraindicated and can be used for coronary calcium scoring in survivors at risk of premature coronary artery disease. Nuclear imaging, such as multigated acquisition scan (MUGA), is now reserved for patients in whom echocardiography and CMR are not feasible^56^.

### 5.2 Biomarkers

Troponin (I or T) and natriuretic peptides (BNP, NT-proBNP) are the principal biomarkers for the detection and monitoring of chemotherapy-induced heart failure. The American Heart Association, American College of Cardiology, and Heart Failure Society of America all recognize the utility of these markers in risk stratification and surveillance for cardiotoxicity in patients receiving agents such as anthracyclines, trastuzumab, and other targeted therapies^59,60^.

The European Society of Cardiology recommends baseline and serial measurement of troponin and natriuretic peptides in high-risk patients, particularly those receiving anthracyclines or trastuzumab^8^.

Elevated levels during or after chemotherapy correlate with increased risk of heart failure and mortality and may be more predictive than LVEF alone for overall death in this population ^60^. BNP and NT-proBNP are sensitive for detecting early LV dysfunction and predicting heart failure events. Serial measurement, especially within 72 hours of chemotherapy and at ~1 month after treatment, can identify patients at risk for developing heart failure before changes in LVEF occur. Persistently elevated troponin is linked to worse prognosis and lack of LVEF recovery^60^.

In summary**,** troponin and natriuretic peptides are the most evidence-based biomarkers for early detection and monitoring of chemotherapy-induced heart failure, with serial measurement recommended in high-risk patients to guide intervention and preserve cardiac function^8,59,60^. This multimodal, risk-stratified approach optimizes early detection of CTRCD, allows timely initiation of cardioprotective therapy, and helps preserve both cardiac and oncologic outcomes.

## 6. Prevention and Management

To effectively mitigate the negative impacts of chemotherapy related to heart failure, a variety of strategies are employed, which include pharmacological interventions, adjustments in chemotherapy delivery, and general recommendations found in cardiovascular prevention protocols. The emergence of heart failure can lead to lasting harm for patients, underscoring the necessity for early and thorough preventive measures.

A thorough initial evaluation, including a detailed medical history, assessment of cardiovascular risk factors, and diagnostic studies such as ECG, echocardiogram, and cardiac biomarkers (troponin, BNP, NT-proBNP), is a key recommendation in clinical practice guidelines for patients who will undergo potentially cardiotoxic therapies, particularly anthracyclines and anti-HER2 agents. The identification and management of comorbidities such as hypertension, obesity, dyslipidemia, and diabetes are essential to reduce the risk of cardiotoxicity^61^.

After completing this evaluation, patients should be categorized based on their cardiovascular risk^55^:

· High risk – individuals on multiple medications (more than five), those taking antiarrhythmics, oral anticoagulants, or tyrosine kinase inhibitors.

· Intermediate risk – cancer patients who are not on multiple medications but have existing heart disease.

· Low risk – patients without any cardiovascular history.

This stratification of risk enables the prediction of possible complications and supports the development of a personalized pharmacological treatment plan.

An additional important measure is to prevent drug interactions, which can greatly elevate the risk of cardiotoxicity. The administration of ACE inhibitors and mineralocorticoid receptor antagonists has shown protective benefits for ventricular function, especially in individuals with raised biomarkers or early indications of ventricular dysfunction. For individuals undergoing anthracycline-based chemotherapy, dexrazoxane is frequently utilized, as it helps decrease the production of free radicals that contribute to heart damage^55^. It is crucial to optimize cardiovascular risk factors, which involves stringent management of blood pressure, glucose, and lipid levels, in addition to lifestyle changes^25^. Moreover, ongoing monitoring throughout and following chemotherapy is essential to identify early signs of cardiac dysfunction, enabling prompt interventions to avert the transition to manifest heart failure.

Managing heart failure is crucial during chemotherapy because many cancer treatments have the potential to damage the heart (cardiotoxic effects). Just like patients without this condition, individuals with existing heart failure need a detailed evaluation by a cardiologist. Those with existing cardiovascular risk factors or who are expected to receive high doses of chemotherapy are considered high-risk. For these patients, it’s recommended to start prophylactic therapy (preventive medication) using drugs like RAAS inhibitors or beta-blockers, as these can help protect the heart muscle^62^. Ongoing monitoring is essential and should include imaging techniques and lab tests, not just clinical assessments. The echocardiogram is the preferred imaging method, mainly used to evaluate the LVEF. However, the most sensitive measure for catching early, subclinical heart muscle damage is the GLS. A relative reduction in GLS of ≥10−15% compared to baseline measurements often happens before a drop in LVEF. This early identification allows for sooner intervention, which can prevent the progression to symptomatic heart failure^63^.

## **7.** Emerging Directions

The modern management of CTRCD is shifting rapidly toward a Precision Cardio-Oncology model, supported by a collaborative, multidisciplinary heart team. At the center of this transition is an emphasis on early and aggressive risk factor modification, particularly targeting the Cardio-Renal-Metabolic Syndrome. Close surveillance and strict blood pressure control are critical, especially in patients receiving vascular-toxic treatments such as TKIs and VEGF inhibitors. Achieving blood pressure levels consistently below 130/80 mmHg is vital to reduce the likelihood of ischemic events and to limit therapy-induced vascular aging^8^.

Emerging pharmacologic strategies are focused on metabolic and homeostatic regulation. SGLT2 inhibitors (such as dapagliflozin and empagliflozin) are gaining particular attention—not only because they have demonstrated reductions in heart failure hospitalizations and mortality across the spectrum of ejection fraction (HFrEF and HFpEF), but also because preliminary evidence suggests they may protect the myocardium from chemotherapy-induced damage. Although early clinical data from observational studies show promise in reducing heart failure hospitalizations, randomized control trials evidence remain limited, with ongoing trials needed to validate efficacy^64-66)^. There are currently no randomized control trials testing SGLT2 inhibitors for preventing chemotherapy-induced cardiotoxicity. The proposed mechanisms of SGLT 2 inhibitors include improved myocardial energy use, reduced oxidative stress, and anti-inflammatory effects. Additionally, chronotherapy, which aligns the timing of anticancer drug administration with the patient’s circadian rhythm, is under investigation as a way to lower cardiac vulnerability and systemic toxicity^66,67^.

A crucial area of development in preventing heart failure in patients undergoing chemotherapy is Predictive Pharmacogenetics. This strategy relies on the use of genomic panels to identify patients with high cardiovascular susceptibility. This risk is determined by searching for Single Nucleotide Polymorphisms (SNPs) in key genes, such as RARG or SLC28A3, which regulate the metabolism of anthracyclines, as well as in myocardial structural genes. Identifying a high-risk genotype not only justifies the early implementation of precision cardioprotective therapies (e.g., Dexrazoxane) but can also influence the selection of an inherently less cardiotoxic oncological regimen from the start of treatment, thus preventing subclinical damage before clinical manifestation^66^. No randomized controlled trials or prospective observational studies specifically examine pharmacogenetic-guided prevention strategies in cardio-oncology populations. The evidence remains at the preclinical and theoretical level, which represents a knowledge gap that requires prospective validation.

Traditional neurohormonal antagonists like ACE inhibitors, ARBs, and beta blockers have mixed randomized controlled trial evidence, with some trials showing LVEF preservation while others demonstrate no significant benefit^64,68^. The STOP-CA trial provided evidence for atorvastatin in preventing LV dysfunction in lymphoma patients receiving anthracyclines representing one of the few positive randomized controlled trials^62^.

## 8. Conclusion

The progress in this medical field, known as Cardio-Oncology, focuses on a multidisciplinary and personalized approach. Strategies such as early patient risk stratification and the implementation of prophylactic cardioprotective therapies are essential for intercepting damage before it progresses to irreversible heart failure. Looking ahead, pharmacogenetics promises to transform the landscape by allowing the identification of individual genetic susceptibilities. This will enable the precise adaptation of both oncologic therapies and cardioprotective protocols. Essentially, the main goal of modern oncology is to optimize cancer survival without sacrificing the patient's long-term quality of life.

## Bullet points

*· Early detection saves hearts: Multimodal surveillance enables identification of subclinical cardiotoxicity before irreversible myocardial damage occurs, allowing timely initiation of cardioprotective therapy while maintaining life-saving cancer treatment.*

*· Precision cardio-oncology is the future: The integration of pharmacogenetic profiling to identify high-risk variants, individualized monitoring protocols, and targeted prophylaxis represents a paradigm shift toward personalized cardiovascular protection that optimizes both cancer survival and long-term quality of life.*
